# Characterization of the gene signature correlated with favorable response to chemoradiotherapy in rectal cancer: A hypothesis‐generating study

**DOI:** 10.1002/cam4.5586

**Published:** 2023-01-09

**Authors:** Seung Hyuck Jeon, Eui Kyu Chie

**Affiliations:** ^1^ Graduate School of Medical Science and Engineering Korea Advanced Institute of Science and Technology Daejeon Korea; ^2^ Department of Radiation Oncology Seoul National University College of Medicine Seoul Korea

**Keywords:** gene signature, immune cell, neoadjuvant chemoradiotherapy, rectal cancer, response

## Abstract

**Purpose:**

This study aimed to define the gene signature associated with response to neoadjuvant chemoradiotherapy (nCRT), or chemoradiosensitivity (CRS) signature, in rectal cancer, and investigate the correlation between the CRS signature and characteristics of tumor.

**Materials and Methods:**

Three public microarray datasets of pre‐nCRT rectal cancer were used to discover and validate the CRS signature, and the pathway analysis of the CRS signature was performed. Patients in The Cancer Genome Atlas (TCGA) dataset were stratified according to the CRS signature enrichment score, and mutational profile and proportions of infiltrated immune cells were compared.

**Results:**

In the discovery dataset (GSE53781), 95 genes were upregulated in complete responders compared to non‐complete responders and defined as the CRS signature. Pathways regarding DNA replication and repair processes as well as inflammatory response were enriched in the CRS signature. In the validation datasets (GSE35452 and GSE45404), patients with favorable response to nCRT exhibited higher enrichment score of the CRS. In TCGA‐READ cohort, patients with high CRS signature harbored *KRAS* mutation in lower frequency than those with low CRS signature. In addition, proportions of proinflammatory immune cells were higher, but proportion of immunosuppressive M2 macrophages was lower in patients with high CRS signature than those with low CRS signature.

**Conclusions:**

The current integrative bioinformatic analysis suggests the CRS signature and showed that the CRS signature is associated with dissimilar mutational profile and increased immune response. The discovered CRS signature and related characteristics may serve as candidate of stratification factor in upcoming studies for rectal cancer.

## Graphical abstract

In this study, we discovered and validated the gene signature associated with response to chemoradiotherapy in rectal cancer, and correlated the gene signature with tumor mutation profile and immune cell infiltration.

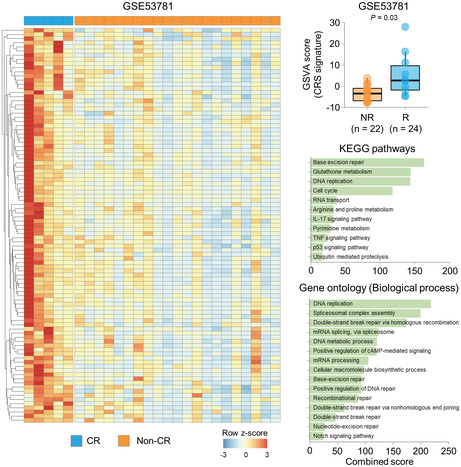

## 1 INTRODUCTION

Neoadjuvant chemoradiotherapy (nCRT) followed by surgical resection is the mainstay of treatment for locally advanced rectal cancer (LARC).^1^ Pathologic response of tumor to nCRT is closely associated with the prognosis of patients;^2^ poor responders to nCRT demonstrate significantly higher rate of locoregional relapse and distant metastasis compared to good responders. On contrary, most patients with complete response to nCRT are free from recurrence, and studies showed that omission of surgical resection may be safe in terms of oncologic outcomes for the patients with clinical complete response.^3^, ^4^ In this context, biomarkers of response to nCRT in rectal cancer are under active investigation.

Several studies reported gene signatures of tumor that are associated with response to nCRT.^5^, ^6^, ^7^, ^8^, ^9^ However, the results are not consistent, leaving the characteristics of nCRT‐sensitive or ‐resistant rectal cancer obscure. Furthermore, most of the previous studies have suggested gene signatures including only a handful of genes to elevate the applicability of the signatures, so that it was difficult to draw biological implication from the gene signatures. Besides gene expression, mutation profiles of tumor are also associated with radiosensitivity.^10^, ^11^ In addition, accumulating evidence implies that tumor microenvironment, including infiltrating immune cells, is another determinant of radiation response.^12^, ^13^, ^14^, ^15^ Although the association between the response to nCRT and various characteristics of tumor or its microenvironment has been extensively explored, none of the features are being used as biomarkers in clinical practice. We hypothesized that studying the relationship between various biological features of tumor in association with response to nCRT would reveal novel insight into radiosensitivity of rectal cancer that would lead to the clinical application.

Here, we analyzed the public datasets of rectal cancer patients who underwent nCRT. We identified the chemoradiosensitivity (CRS) signature and validated the signature in the independent datasets. Additionally, we characterized the tumor mutation profiles and immune cell infiltration according to the enrichment of the CRS signature and identified novel features of nCRT‐sensitive rectal cancer.

## 2 MATERIALS AND METHODS

### 2.1 Public datasets

Publicly available microarray datasets, of which processed matrices were available, were selected for analysis in this study. Preprocessed matrices of three datasets (GSE53781,^8^ GSE35452,^16^ and GSE45404^17^) were downloaded from the Gene Expression Omnibus database (http://www.ncbi.nlm.nih.gov/geo). The preprocessed matrices included the normalized expression values for each probe. Then, the probe sets of CodeLink Human Whole Genome Array and Affymetrix Human Genome U133 Plus 2.0 Array were mapped to gene symbols using R packages *hwgcodSYMBOL* and *hgu133plus2SYMBOL*, respectively. For probe sets representing a single gene, the probes with the highest expression value were selected. Additional correction was not used because the two microarray platforms were reported to be highly correlated.^18^ All patients in the datasets underwent concurrent 5‐fluorouracil (5‐FU)‐based chemotherapy with radiotherapy. The characteristics of the three datasets are detailed in Table 1. Additionally, mRNA‐sequencing (mRNA‐seq) data of rectal adenocarcinoma in The Cancer Genome Atlas (TCGA‐READ) database was also analyzed. The clinical information and RSEM values of total transcripts were downloaded from the Broad Institute GDAC Firehose (https://gdac.broadinstitute.org).

**TABLE 1:** Details of public microarray datasets analyzed in the study

Dataset	Platform	Patient no.	Non‐responder	Responder
GSE53781	CodeLink Human Whole Genome Array	26	16	10
GSE35452	Affymetrix Human Genome U133 Plus 2.0 Array	46	22	24
GSE45404	Affymetrix Human Genome U133 Plus 2.0 Array	42	23	19

### 2.2 Definition and characterization of gene signature

Based on the normalized expression, the empirical Bayes method was used to perform differential expression analysis in an R package *limma*. Differentially expressed genes (DEGs) were determined as log2 fold change >2 and false discovery rate <0.1. The genes upregulated in tumor of complete responders were defined as the gene signature of complete responder, or CRS signature. Enrichment score of the gene signature in each sample was calculated using gene set variation analysis (GSVA), with the combined *z*‐score method and Gaussian kernel. The pathway analysis of the gene signatures was performed using gene list enrichment analysis tool, *Enrichr*; the Kyoto Encyclopedia of Genes and Genomes (KEGG) pathways and Gene ontology pathways were analyzed. The combined score was calculated as a parameter of enrichment as the log (*p*‐value) multiplied by the *z*‐score from the Fisher exact test.

### 2.3 TCGA dataset analysis

Among the patients in TCGA‐READ dataset, patients without mRNA‐seq data or mutation profiles were excluded from the analysis. Additionally, unlike the microarray datasets, patients who received neoadjuvant therapy were excluded since neoadjuvant therapy may alter the baseline characteristics of tumor. Finally, 85 patients were available for the analysis. The patients were divided into two groups, patients with low and high CRS signature, dichotomized using the median enrichment score. The data regarding tumor mutational burden were downloaded from the previous publication.^19^ Composition of tumor‐infiltrating immune cells was estimated using a deconvolution algorithm CIBERSORT.^20^

### 2.4 Statistical analysis

The continuous variables and categorical variables between two groups were compared using Student's *t*‐test and chi‐squared test, respectively. AUC values of the CRS signature scores were calculated using an R package *pROC*. The Kaplan–Meier curves of survival were compared using Cox‐proportional hazard model. *p*‐value <0.05 was considered statistically significant. All statistical analyses were performed with R software v.4.1.0 (https://www.r‐project.org).

## 3 RESULTS

### 3.1 Discovery and biological characterization of the CRS signature

**FIGURE 1:**
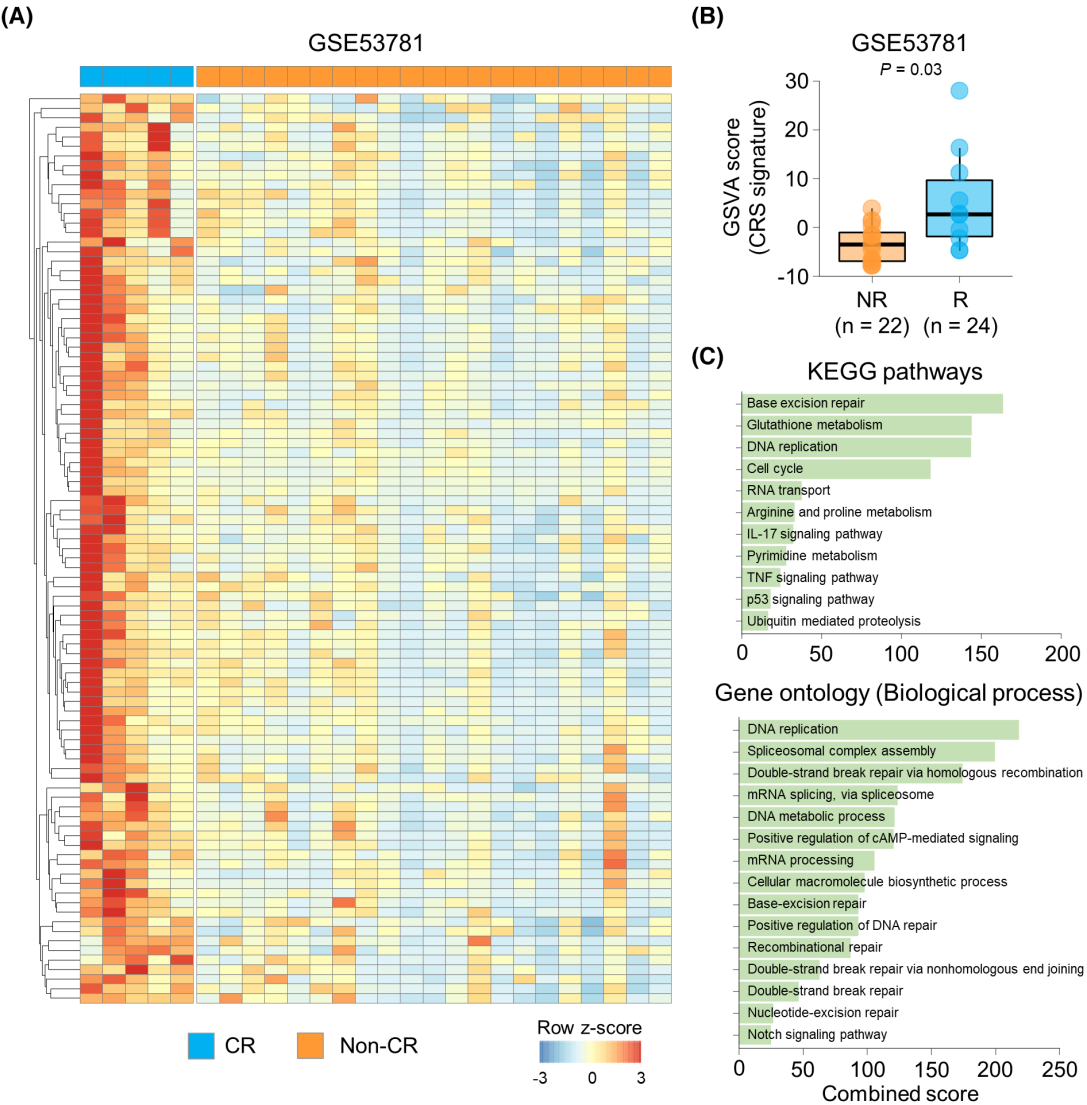
Identification and characterization of the chemoradiosensitivity (CRS) signature. (A) Heatmap of the expression pattern of the differentially expressed genes between complete responders and non‐complete responders, defined as the CRS signature, in the GSE53781 dataset. (B) The gene set variation analysis score of the CRS signature in patients with non‐response (tumor regression grade [TRG] ≥ 3) and response (TRG ≤2). *P*‐value was calculated using *t*‐test. (C) Combined scores of KEGG pathways (top) and Gene ontology (bottom) with statistical significance for the CRS signature.

We first discovered the gene signature of good response in the dataset of GSE53781 that stratified patients using 5‐tier tumor regression grade (TRG). Among the spectrum of tumor response, it is believed that tumors showing complete response would exhibit the features of good responding tumors in the greatest extent. Therefore, we compared the gene expression profiles between complete responders (TRG = 1) and non‐complete responders (TRG ≥ 2). We identified 95 upregulated DEGs in complete responders (Figure 1A and Table S1) and defined them as the CRS signature. When we broadened the definition of responder (TRG ≤ 2), enrichment score of the CRS signature was significantly higher in the responders than non‐responders (*p* = 0.027; Figure 1B). Furthermore, the CRS signature score predicted the response to nCRT with AUC value of 0.794 (*p* = 0.002; Figure S1).

To characterize the biological process associated with good response, we carried out pathway analysis of and KEGG pathways in the Gene ontology (Figure 1C; Tables S2 and S3). We found that DNA replication and repair processes were highly enriched in the CRS signature. Signaling pathways associated with inflammatory response, including IL‐17 (*p* = 0.010) and TNF signaling pathways (*p* = 0.016), were also enriched in the CRS signature. In addition, Notch signaling pathway (*p* = 0.033) was over‐represented in the CRS signature. Collectively, the CRS signature provided insights into the biological features of rectal cancer that exhibits good response to nCRT.

### 3.2 Validation of the CRS signature in independent datasets

**FIGURE 2:**
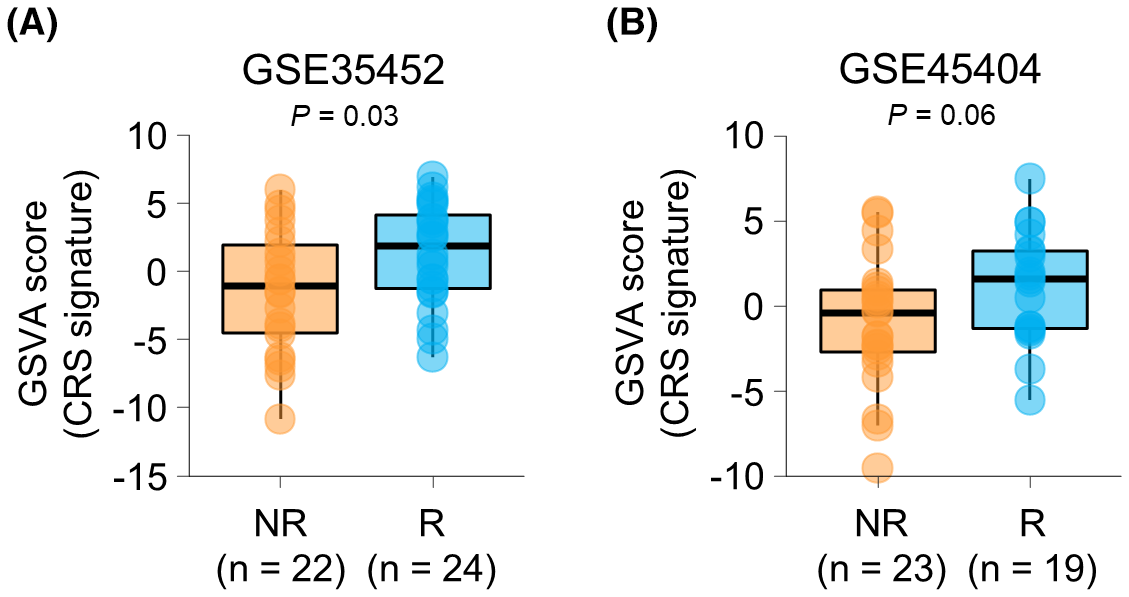
Validation of the chemoradiosensitivity (CRS) signature. (A, B) The gene set variation analysis score of the CRS signature in patients with non‐response and response in GSE35452 (A) and GSE45404 datasets (B). *p*‐values were calculated using *t*‐test.

To validate the predictive role of the CRS signature in independent datasets, we used two public microarray datasets (GSE35452 and GSE45404). In GSE35452, the responders had significantly higher enrichment score of the CRS signature compared to non‐responders (*p* = 0.032, Figure 2A). Similarly, responders showed greater enrichment of the CRS signature than non‐responders with marginal statistical significance in GSE45404 (*p* = 0.061; Figure 2B). Moreover, the AUC values for predictability of response using enrichment score of CRS signature were 0.674 (*p* = 0.03) and 0.675 (*p* = 0.04) in GSE35452 and GSE45404 datasets, respectively. Together, these results indicate that the discovered CRS signature may work as a universal gene signature to predict response of rectal cancer to nCRT.

### 3.3 Association between the CRS signature and mutation profiles of cancer

To obtain more insight into the characteristics of tumor according to the enrichment of the CRS signature, we analyzed the 85 patients in TCGA‐READ dataset who did not undergo neoadjuvant therapy. Patients were stratified by the enrichment score of the CRS signature. The baseline characteristics of patients are detailed in Table 2. Patients with high CRS signature were significantly younger than those with low CRS signature (60.2 vs. 65.7 years, *p* = 0.042). Stage of disease was not significantly different between the two groups. The progression‐free survival (*p* = 0.27) and overall survival (*p* = 0.07) were not significantly different according to the CRS signature (Figure S2), suggesting that the signature is predictive rather than prognostic.

**TABLE 2:** Characteristics of patients in TCGA‐READ database with high and low CRS signature

Characteristics	CRS signature‐high (*n* = 42)	CRS signature‐low (*n* = 43)	*p*‐value
Age, mean (± *SD*)	60.2 ± 12.9	65.7 ± 11.9	0.042^a^
Sex			0.325^b^
Male	25 (59.5%)	20 (46.5%)	
Female	17 (40.5%)	23 (53.5%)	
Stage			0.446^b^
I	8 (19.5%)	4 (10.0%)	
II	11 (26.8%)	14 (35.0%)	
III	17 (41.5%)	14 (35.0%)	
IV	5 (12.2%)	8 (20.0%)	
T stage			0.255^b^
T1‐2	11 (26.2%)	6 (14.0%)	
T3‐4	31 (73.8%)	37 (86.0%)	
N stage			1.000^b^
N0	19 (45.2%)	20 (47.6%)	
N1‐2	23 (54.8%)	22 (52.4%)	
Distant metastasis			1.000^b^
Absent	33 (86.8%)	30 (85.7%)	
Present	5 (13.2%)	5 (14.3%)	

Abbreviations: CRS, chemoradiosensitivity; *SD*, standard deviation.

^a^ Student's *t*‐test.

^b^ Chi‐squared test.

We found that total mutation count (466.1 vs. 141.7, *p* = 0.23) and tumor mutational burden (15.4 vs. 4.7 per 10^6^ bp, *p* = 0.23) were numerically higher in patients with high CRS signature compared to those with low CRS signature, but the difference was not statistically significant. When we categorized the type of mutation, neither synonymous (3.2 vs. 1.2 per 10^6^ bp, *p* = 0.25) nor non‐synonymous (12.2 vs. 3.5 per 10^6^ bp, *p* = 0.23) mutational burden was not significantly different between patients with high and low CRS signature.

Next, we compared the mutation profiles of rectal cancer according to the enrichment of the CRS signature in TCGA database. We analyzed the 30 most commonly mutated genes in the dataset, including *APC*, *TP53*, *KRAS*, *PI3KCA*, *SMAD4*, and *NRAS*. Among the analyzed genes, *KRAS* mutation was less frequent in patients with high CRS signature (23.8% vs. 48.8%, *p* = 0.030) than those with low CRS signature. In patients with high CRS signature, G13D mutation (3/10) was the most common, followed by G12V mutation (2/10). On the other hand, G12V mutation (8/21) was the most common oncogenic mutation in the patients with low CRS signature, followed by G12D (5/21) and G13D (4/21) mutations. No patients in the high CRS signature group harbored *KRAS* G12D mutation. In addition, patients with high CRS signature were more likely to harbor *APC* mutation (95.2% vs. 79.1%, *p* = 0.058) with marginal statistical significance. Mutation of other genes were not significantly different between the two groups. Comparisons on somatic mutation profiles between the two groups are summarized on Table 3.

**TABLE 3:** Mutation profile of patients in TCGA‐READ database with high and low CRS signature

Gene	Total (*n* = 85)	CRS signature‐high (*n* = 42)	CRS signature‐low (*n* = 43)	*p*‐value
APC	74 (87.1%)	40 (95.2%)	34 (79.1%)	0.058
TP53	73 (85.9%)	39 (92.9%)	34 (79.1%)	0.130
TTN	37 (43.5%)	16 (38.1%)	21 (48.8%)	0.435
KRAS	31 (36.5%)	10 (23.8%)	21 (48.8%)	0.030
SYNE1	24 (28.2%)	12 (28.6%)	12 (27.9%)	1.000
RYR2	18 (21.2%)	10 (23.8%)	8 (18.6%)	0.748
FLG	17 (20.0%)	8 (19.0%)	9 (20.9%)	1.000
LRP1B	16 (18.8%)	9 (21.4%)	7 (16.3%)	0.742
MUC16	16 (18.8%)	8 (19.0%)	8 (18.6%)	1.000
FAT4	14 (16.5%)	10 (23.8%)	4 (9.3%)	0.131
FBXW7	14 (16.5%)	9 (21.4%)	5 (11.6%)	0.355
FAT3	13 (15.3%)	6 (14.3%)	7 (16.3%)	1.000
CXMD1	13 (15.3%)	4 (9.5%)	9 (20.9%)	0.246
UNC80	13 (15.3%)	7 (16.7%)	6 (14.0%)	0.963
HYDIN	12 (14.1%)	9 (21.4%)	3 (7.0%)	0.109
DNAH5	11 (12.9%)	8 (19.0%)	3 (7.0%)	0.182
PEG3	11 (12.9%)	5 (11.9%)	6 (14.0%)	1.000
SMAD4	11 (12.9%)	3 (7.1%)	8 (18.6%)	0.211
NEB	11 (12.9%)	6 (14.3%)	5 (11.6%)	0.967
DNAH11	11 (12.9%)	4 (9.5%)	7 (16.3%)	0.546
RYR1	11 (12.9%)	8 (19.0%)	3 (7.0%)	0.182
NRAS	11 (12.9%)	6 (14.3%)	5 (11.6%)	0.967
PIK3CA	10 (11.8%)	6 (14.3%)	4 (9.3%)	0.707
SACS	10 (11.8%)	6 (14.3%)	4 (9.3%)	0.707
OBSCN	10 (11.8%)	5 (11.9%)	5 (11.6%)	1.000
ATM	10 (11.8%)	6 (14.3%)	4 (9.3%)	0.707
UNC13C	9 (10.6%)	4 (9.5%)	5 (11.6%)	1.000
COL6A3	9 (10.6%)	5 (11.9%)	4 (9.3%)	0.970
PKHD1	9 (10.6%)	5 (11.9%)	4 (9.3%)	0.970
USH2A	8 (9.4%)	4 (9.5%)	4 (9.3%)	1.000
XIRP2	8 (9.4%)	5 (11.9%)	3 (7.0%)	0.684
SPTA1	8 (9.4%)	4 (9.5%)	4 (9.3%)	1.000
CSMD3	7 (8.2%)	4 (9.5%)	3 (7.0%)	0.974
ZFHX4	6 (7.1%)	3 (7.1%)	3 (7.0%)	1.000

*Note*: *P*‐values were calculated using the chi‐squared test.

Abbreviation: CRS, chemoradiosensitivity.

### 3.4 Association between the CRS signature and immune cell infiltration into cancer

**FIGURE 3:**
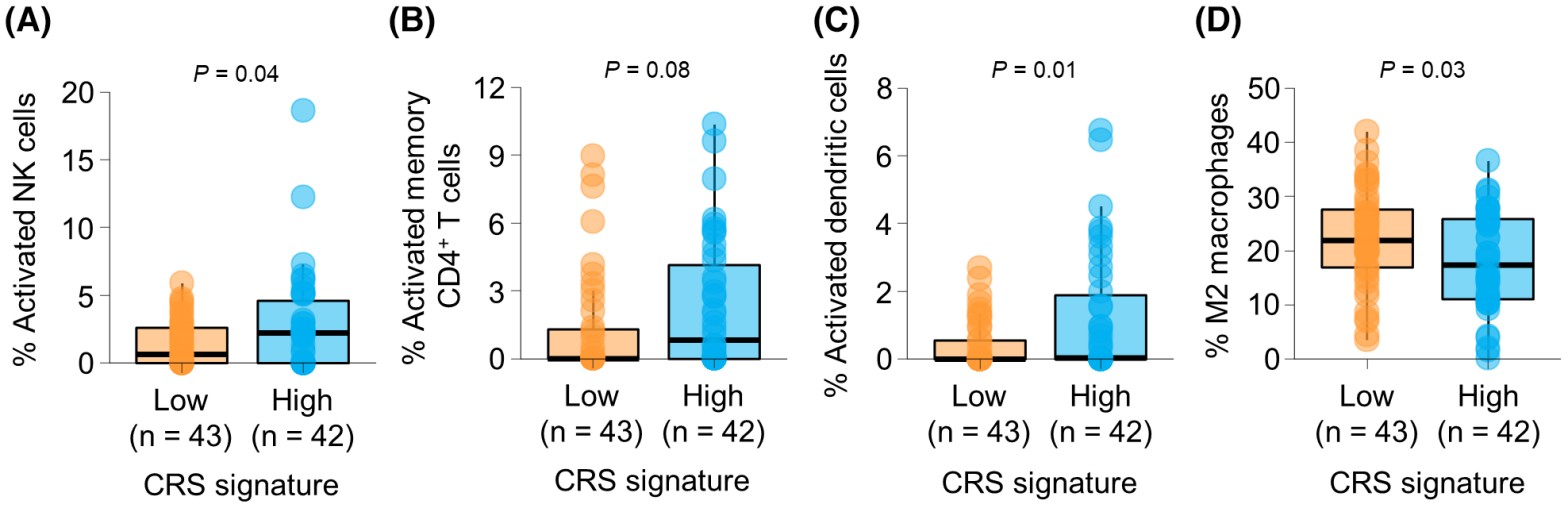
Association between proportion of tumor‐infiltrating immune cells and the chemoradiosensitivity (CRS) signature. (A–D) The proportion of activated NK cells (A), activated memory CD4^+^ T cells (B), activated dendritic cells (C), and M2 macrophages (D), estimated by CIBERSORT, according to the enrichment of the CRS signature in TCGA‐READ cohort. *p*‐values were calculated using *t*‐test.

We next examined the proportions of tumor‐infiltrating immune cells by deconvolution of RNA‐seq data in TCGA dataset (Table 4). Activated NK cells were significantly more frequent in tumor with high CRS signature compared to low CRS signature (2.80% vs. 1.48%, *p* = 0.038; Figure 3A). The proportion of activated memory CD4^+^ T cells was marginally higher in tumor with high CRS signature compared to low CRS signature (2.29% vs. 1.26%, *p* = 0.075; Figure 3B). Activated dendritic cells were also significantly more abundant in tumor with high CRS signature than low CRS signature (1.20% vs. 0.40%, *p* = 0.011; Figure 3C). On contrary, the proportion of immunosuppressive M2 macrophages was significantly lower in tumor with high CRS signature than low CRS signature (17.91% vs. 22.38%, *p* = 0.026; Figure 3D). Other immune cell subsets, including cytotoxic CD8^+^ T cells and regulatory CD4^+^ T cells, populated similarly in the two groups.

**TABLE 4:** Estimated proportions of immune cells in TCGA‐READ cohort with high and low CRS signature

Immune cell subset	CRS signature‐high (*n* = 42)	CRS signature‐low (*n* = 43)	*P*‐value
Naive B cells, %	4.34 ± 5.08	3.72 ± 3.33	0.506
Memory B cells, %	1.99 ± 3.87	1.92 ± 3.77	0.931
Plasma cells, %	4.09 ± 3.15	3.77 ± 4.51	0.696
CD8^+^ T cells, %	8.23 ± 4.42	9.01 ± 4.88	0.443
Naive CD4^+^ T cells, %	0.50 ± 2.75	0.44 ± 2.08	0.910
Resting memory CD4^+^ T cells, %	15.92 ± 8.76	14.80 ± 9.80	0.578
Activated memory CD4^+^ T cells, %	2.29 ± 2.88	1.26 ± 2.39	0.075
Follicular helper CD4+ T cells, %	6.31 ± 4.05	5.73 ± 3.56	0.489
Regulatory T cells, %	2.37 ± 2.38	2.87 ± 2.29	0.325
Resting NK cells, %	2.80 ± 3.64	2.06 ± 2.57	0.284
Activated NK cells, %	2.80 ± 3.68	1.48 ± 1.68	0.038
Monocytes, %	1.58 ± 1.99	2.12 ± 2.03	0.217
M0 macrophages, %	14.04 ± 8.01	14.67 ± 12.00	0.779
M1 macrophages, %	5.51 ± 4.06	4.89 ± 4.06	0.486
M2 macrophages, %	17.91 ± 9.15	22.38 ± 9.07	0.026
Resting dendritic cells, %	0.91 ± 2.31	1.43 ± 1.94	0.261
Activated dendritic cells, %	1.20 ± 1.82	0.40 ± 0.72	0.011
Resting mast cells, %	1.40 ± 2.76	1.92 ± 2.69	0.385
Activated mast cells, %	4.24 ± 4.93	4.23 ± 5.11	0.990
Eosinophils, %	0.42 ± 1.41	0.30 ± 0.90	0.646
Neutrophils, %	1.12 ± 3.11	0.61 ± 1.66	0.344

*Note*: *p*‐values were calculated using Student's *t*‐test.

Abbreviation: CRS, chemoradiosensitivity.

## 4 DISCUSSION

This bioinformatic analysis of public database showed the correlation between the gene signature associated with response to nCRT in multiple datasets and various biological characteristics of rectal cancer. We identified that somatic mutation of specific genes and proportion of infiltrating immune cells are associated with response to nCRT in rectal cancer. To the best of our knowledge, this is the first study to comprehensively analyze the factors, including expression of specific pathways, somatic mutations of cancer, and infiltration of immune cells, associated with the response of rectal cancer to nCRT. Whether the response of rectal cancer to nCRT is associated with the somatic mutations of *KRAS* or *APC* and infiltration of activated CD4^+^ T cells, NK cells, dendritic cells, or M2 macrophages should be verified in future investigations.

Our CRS signature included several genes that were reported in the previous studies. Along with *GNG4*, *MYC*, and *RRM1*, which were included in the four‐gene signature derived from GSE53781, the discovery dataset of our study, *BRCA1*, which was included in the four‐gene signature in the previous study,^21^ was included the CRS signature. The characterization of the signature validated in independent datasets showed that nCRT‐sensitive tumors are enriched for pathways associated with DNA replication and repair processes. It is well accepted that DNA repair pathways are associated with cancer therapeutic resistance, including radiation and 5‐FU,^22^, ^23^ which is opposite to our findings. However, it can be postulated that downregulation of DNA repair pathways may confer increased somatic mutations, some of which are associated with sensitivity to nCRT, supported by the numerically higher mutation load in patients with high CRS signature. In addition, the genes involved in Notch signaling pathway were enriched in radiosensitive rectal cancer. We have previously shown the association between the genomic alteration of Notch signaling pathway‐associated genes and response to radiation in various cancer types.^24^, ^25^ Furthermore, the association between sensitivity to 5‐FU and Notch signaling pathway has been also proposed.^26^ Further studies are needed to examine the association between various molecular pathways and sensitivity to nCRT of rectal cancer.

Our results showed that somatic mutation of *KRAS* may be associated with low likelihood of good response to nCRT consistent with the results from previous studies.^10^, ^11^ The *KRAS* G12D mutation was exclusively observed in the low CRS signature group. Moreover, the most common mutation type was *KRAS* G13D in high CRS signature group, while it was *KRAS* G12V in low CRS signature group. Accumulating evidence indicates the distinct therapeutic responses of tumor with specific *KRAS* mutation^27^; for example, *KRAS* G13D mutation is associated with sensitivity to anti‐EGFR therapies.^28^ The association between *KRAS* G12D mutation and low CRS signature in current study may have stemmed from the limited response to chemotherapy in *KRAS* G12D mutation harboring colorectal cancer,^29^ although the relationship between specific KRAS mutation status and response to radiotherapy is largely unknown. The previous works also showed that mutation of *TP53* was associated with radioresistance of rectal cancer, which was not demonstrated in TCGA dataset. Additionally, somatic mutation of *APC* was marginally associated with chemoradiosensitivity of rectal cancer. No preclinical or human studies have shown the association between somatic mutation of *APC* in cancer cells and their sensitivity to nCRT. The effect of different *KRAS* mutations and *APC* mutation on sensitivity to nCRT should be investigated in the future studies.

There have been several reports that showed the association between infiltrating immune cells and response to nCRT in rectal cancer. Previous works showed that increased infiltrating of T cells, especially CD8^+^ T cells, is associated with favorable response to nCRT.^13^, ^14^, ^15^ Our results did not exhibit significant difference of CD8^+^ T cell infiltration in patients with high and low CRS signature; however, higher proportions of activated memory CD4^+^ T cells, activated NK cells, and activated dendritic cells, which are thought to play anti‐tumor role in tumor microenvironment, were observed in patients with high CRS signature. Conversely, immunosuppressive M2 macrophages were less frequent in patients with high CRS signature, indicating that anti‐tumor response against tumor cells may be associated with sensitivity of rectal cancer to nCRT. Enrichment of inflammatory pathways in CRS signature further supports these findings. CD4^+^ T cells consist of various subsets, and a previous study showed that higher Th1 subset and lower Th17 subset are associated with favorable prognosis in colorectal cancer.^30^ Therefore, deeper characterization of the immune cells in regard to tumor response is necessary.

Current analysis is not free of innate limitations. Because patients in all three microarray datasets underwent concurrent 5‐FU‐based chemotherapy, we were unable to separate the effect of 5‐FU and radiotherapy. Furthermore, although enrichment of the CRS signature was validated in two independent datasets, the signature was derived from the upregulated genes in complete responders; however, the validation datasets did not provide the information regarding the complete response. Since the signature may be enriched only in tumor with remarkable chemoradiosensitivity, the signature should be compared in patients who did and did not experience complete response. In addition, the deconvolution method has limited ability to estimate the proportion of immune cells, because it is based on the reference gene expression profiles, which could deviate in cells within tumor microenvironment.^20^ Accurate enumeration of immune cells should be conducted to validate the results of current study. The stratification of patients in TCGA database was performed according to the CRS signature, not the actual response to nCRT. Although the signature was validated in the independent datasets, the stratification based on the signature may bring about potential bias; thus, the results should be carefully interpreted. Therefore, it might be helpful to verify the CRS signature and its association with other characteristics using primary multiomic data.

In conclusion, this integrative bioinformatic analysis provided novel biological insight into the response of rectal cancer to nCRT. We believe that the new features may be utilized in combination with other previously described biomarkers to predict the response. Validation of these findings and investigation on the mechanisms underlying the association should be conducted in future works.

## AUTHOR CONTRIBUTIONS

**Seung Hyuck Jeon:** Conceptualization (equal); data curation (equal); formal analysis (equal); funding acquisition (equal); investigation (equal); methodology (equal); project administration (equal); resources (equal); software (equal); supervision (equal); validation (equal); visualization (equal); writing – original draft (equal); writing – review and editing (equal). **Eui Kyu Chie:** Conceptualization (equal); data curation (equal); formal analysis (equal); funding acquisition (equal); investigation (equal); methodology (equal); project administration (equal); resources (equal); software (equal); supervision (equal); validation (equal); visualization (equal); writing – original draft (equal); writing – review and editing (equal).

## CONFLICT OF INTEREST

The authors have no conflict of interest.

## Supporting information


Table S1.
Table S2.
Table S3.

Figure S1.
Figure S2.

## Data Availability

The data that support the findings of this study are openly accessible through the Gene Expression Omnibus (GEO) database.

## References

[1] Sauer R , Liersch T , Merkel S , et al. Preoperative versus postoperative chemoradiotherapy for locally advanced rectal cancer: results of the German CAO/ARO/AIO‐94 randomized phase III trial after a median follow‐up of 11 years. J Clin Oncol. 2012;30(16):1926‐1933. doi:10.1200/JCO.2011.40.1836 22529255

[2] Fokas E , Liersch T , Fietkau R , et al. Tumor regression grading after preoperative chemoradiotherapy for locally advanced rectal carcinoma revisited: updated results of the CAO/ARO/AIO‐94 trial. J Clin Oncol. 2014;32(15):1554‐1562. doi:10.1200/JCO.2013.54.3769 24752056

[3] van der Valk MJM , Hilling DE , Bastiaannet E , et al. Long‐term outcomes of clinical complete responders after neoadjuvant treatment for rectal cancer in the International Watch & Wait Database (IWWD): an international multicentre registry study. Lancet. 2018;391(10139):2537‐2545. doi:10.1016/S0140-6736(18)31078-X 29976470

[4] Renehan AG , Malcomson L , Emsley R , et al. Watch‐and‐wait approach versus surgical resection after chemoradiotherapy for patients with rectal cancer (the OnCoRe project): a propensity‐score matched cohort analysis. Lancet Oncol. 2016;17(2):174‐183. doi:10.1016/S1470-2045(15)00467-2 26705854

[5] Ghadimi BM , Grade M , Difilippantonio MJ , et al. Effectiveness of gene expression profiling for response prediction of rectal adenocarcinomas to preoperative chemoradiotherapy. J Clin Oncol. 2005;23(9):1826‐1838. doi:10.1200/JCO.2005.00.406 15774776 PMC4721601

[6] Kim IJ , Lim SB , Kang HC , et al. Microarray gene expression profiling for predicting complete response to preoperative chemoradiotherapy in patients with advanced rectal cancer. Dis Colon Rectum. 2007;50(9):1342‐1353. doi:10.1007/s10350-007-277-7 17665260

[7] Brettingham‐Moore KH , Duong CP , Greenawalt DM , et al. Pretreatment transcriptional profiling for predicting response to neoadjuvant chemoradiotherapy in rectal adenocarcinoma. Clin Cancer Res. 2011;17(9):3039‐3047. 21224373 10.1158/1078-0432.CCR-10-2915

[8] Palma P , Cano C , Conde‐Muiño R , et al. Expression profiling of rectal tumors defines response to neoadjuvant treatment related genes. PLoS One. 2014;9(11):e112189. doi:10.1371/JOURNAL.PONE.0112189 25380052 PMC4224421

[9] Casado E , García VM , Sánchez JJ , et al. A combined strategy of SAGE and quantitative PCR provides a 13‐gene signature that predicts preoperative chemoradiotherapy response and outcome in rectal cancer. Clin Cancer Res. 2011;17(12):4145‐4154. 21467161 10.1158/1078-0432.CCR-10-2257

[10] Salnikova LE , Kolobkov DS . Germline and somatic genetic predictors of pathological response in neoadjuvant settings of rectal and esophageal cancers: systematic review and meta‐analysis. Pharmacogenomics J. 2016;16(3):249‐265. doi:10.1038/tpj.2015.46 26122021

[11] Kamran SC , Lennerz JK , Margolis CA , et al. Integrative molecular characterization of resistance to neoadjuvant chemoradiation in rectal cancer. Clin Cancer Res. 2019;25(18):5561‐5571. doi:10.1158/1078-0432.CCR-19-0908 31253631 PMC6744983

[12] Nicolas AM , Pesic M , Engel E , et al. Inflammatory fibroblasts mediate resistance to neoadjuvant therapy in rectal cancer. Cancer Cell. 2022;40(2):168‐184. doi:10.1016/j.ccell.2022.01.004 35120600

[13] Yasuda K , Nirei T , Sunami E , Nagawa H , Kitayama J . Density of CD4(+) and CD8(+) T lymphocytes in biopsy samples can be a predictor of pathological response to chemoradiotherapy (CRT) for rectal cancer. Radiat Oncol. 2011;6(1):1‐6. doi:10.1186/1748-717X-6-49 21575175 PMC3120676

[14] Akiyoshi T , Gotoh O , Tanaka N , et al. T‐cell complexity and density are associated with sensitivity to neoadjuvant chemoradiotherapy in patients with rectal cancer. Cancer Immunol Immunother. 2021;70(2):509‐518. doi:10.1007/s00262-020-02705-6 32845355 PMC10991251

[15] El SC , Kirilovsky A , van den Eynde M , et al. A diagnostic biopsy‐adapted Immunoscore predicts response to neoadjuvant treatment and selects patients with rectal cancer eligible for a watch‐and‐wait strategy. Clin Cancer Res. 2020;26(19):5198‐5207. doi:10.1158/1078-0432.CCR-20-0337 32669377

[16] Watanabe T , Kobunai T , Akiyoshi T , Matsuda K , Ishihara S , Nozawa K . Prediction of response to preoperative chemoradiotherapy in rectal cancer by using reverse transcriptase polymerase chain reaction analysis of four genes. Dis Colon Rectum. 2014;57(1):23‐31. doi:10.1097/01.dcr.0000437688.33795.9d 24316942

[17] Agostini M , Zangrando A , Pastrello C , et al. A functional biological network centered on XRCC3: a new possible marker of chemoradiotherapy resistance in rectal cancer patients. Cancer Biol Ther. 2015;16(8):1160‐1171. doi:10.1080/15384047.2015.1046652 26023803 PMC4622011

[18] Yauk CL , Berndt ML , Williams A , Douglas GR . Comprehensive comparison of six microarray technologies. Nucleic Acids Res. 2004;32(15):e124. doi:10.1093/NAR/GNH123 15333675 PMC516080

[19] Thorsson V , Gibbs DL , Brown SD , et al. The immune landscape of cancer. Immunity. 2018;48(4):812‐830. 29628290 10.1016/j.immuni.2018.03.023 PMC5982584

[20] Newman AM , Liu CL , Green MR , et al. Robust enumeration of cell subsets from tissue expression profiles. Nat Methods. 2015;12(5):453‐457. doi:10.1038/nmeth.3337 25822800 PMC4739640

[21] Momma T , Okayama H , Kanke Y , et al. Validation of gene expression‐based predictive biomarkers for response to neoadjuvant chemoradiotherapy in locally advanced rectal cancer. Cancers. 2021;13(18):4642. doi:10.3390/cancers13184642 34572869 PMC8467397

[22] Li LY , Di GY , Chen XS , Yang JM , Cheng Y . DNA repair pathways in cancer therapy and resistance. Front Pharmacol. 2021;11:2520. 10.3389/fphar.2020.629266 PMC7898236 33628188

[23] Sethy C , Kundu CN . 5‐fluorouracil (5‐FU) resistance and the new strategy to enhance the sensitivity against cancer: implication of DNA repair inhibition. Biomed Pharmacother. 2021;137:111285. doi:10.1016/J.BIOPHA.2021.111285 33485118

[24] Jeon SH , Chie EK , Kim YJ , et al. Targeted next‐generation DNA sequencing identifies notch signaling pathway mutation as a predictor of radiation response. Int J Radiat Biol. 2019;95(12):1640‐1647. doi:10.1080/09553002.2019.1665212 31525117

[25] Jang B‐S , Chang J‐H , Jeon SH , et al. Radiation response prediction model based on integrated clinical and genomic data analysis. Cancer Res Treat. 2022;54(2):383‐395. doi:10.4143/crt.2021.759 34425668 PMC9016297

[26] Ghafouri‐Fard S , Abak A , Tondro Anamag F , et al. 5‐fluorouracil: a narrative review on the role of regulatory mechanisms in driving resistance to this chemotherapeutic agent. Front Oncol. 2021;11:1210. doi:10.3389/FONC.2021.658636/BIBTEX PMC8092118 33954114

[27] Zhu G , Pei L , Xia H , Tang Q , Bi F . Role of oncogenic KRAS in the prognosis, diagnosis and treatment of colorectal cancer. Mol Cancer. 2021;20:143. doi:10.1186/S12943-021-01441-4 34742312 PMC8571891

[28] De Roock W , Jonker DJ , Di Nicolantonio F , et al. Association of KRAS p.G13D mutation with outcome in patients with chemotherapy‐refractory metastatic colorectal cancer treated with cetuximab. Jama. 2010;304(16):1812‐1820. doi:10.1001/JAMA.2010.1535 20978259

[29] Zocche DM , Ramirez C , Fontao FM , Costa LD , Redal MA . Global impact of KRAS mutation patterns in FOLFOX treated metastatic colorectal cancer. Front Genet. 2015;6:116. doi:10.3389/FGENE.2015.00116/BIBTEX 25870609 PMC4378307

[30] Tosolini M , Kirilovsky A , Mlecnik B , et al. Clinical impact of different classes of infiltrating T cytotoxic and helper cells (Th1, Th2, Treg, Th17) in patients with colorectal cancer. Cancer Res. 2011;71(4):1263‐1271. doi:10.1158/0008-5472.CAN-10-2907 21303976

