# Supplementary material for: Characterization of the gene signature correlated with favorable response to chemoradiotherapy in rectal cancer: A hypothesis‐generating study
Source: Cancer Med. 2023 Jan 9;12(7):8981–90. doi: 10.1002/cam4.5586 (PMC10134325; doi:10.1002/cam4.5586)
Supplement: Supplementary file 1 — Table S1. Table S2. Table S3. [file CAM4-12-8981-s001.docx]

**Supplementary Table S1.** List of differentially expressed genes upregulated in complete responders in GSE53781 dataset.

| Gene | Log2(fold change) | False discovery rate |
| --- | --- | --- |
| UCA1 | 65.11184 | 0.026982 |
| CXCL3 | 48.5082 | 0.058564 |
| GEMIN6 | 18.38523 | 0.058564 |
| POFUT1 | 8.229829 | 0.058564 |
| ERMARD | 5.707487 | 0.07642 |
| FAT1 | 35.43839 | 0.07642 |
| GNG4 | 28.13209 | 0.07642 |
| JUN | 2.474733 | 0.07642 |
| MCM10 | 9.778545 | 0.07642 |
| MRTO4 | 14.83147 | 0.07642 |
| STMN1 | 9.270534 | 0.07642 |
| C11orf58 | 25.7128 | 0.077283 |
| CHAF1A | 16.26841 | 0.077283 |
| CRCP | 2.22134 | 0.077283 |
| CSE1L | 145.5591 | 0.077283 |
| CSTF1 | 31.11635 | 0.077283 |
| DDX55 | 6.957335 | 0.077283 |
| FEN1 | 103.8472 | 0.077283 |
| FIBIN | 3.763542 | 0.077283 |
| IL34 | 3.825715 | 0.077283 |
| IPO7 | 10.24562 | 0.077283 |
| MCM6 | 8.291598 | 0.077283 |
| MRPL11 | 17.74595 | 0.077283 |
| MSH6 | 14.60059 | 0.077283 |
| MYC | 107.0547 | 0.077283 |
| NEU1 | 28.55521 | 0.077283 |
| NPVF | 5.783045 | 0.077283 |
| PRPF19 | 5.775856 | 0.077283 |
| PRSS23 | 6.133363 | 0.077283 |
| RRM2 | 26.30369 | 0.077283 |
| SUV39H1 | 12.87447 | 0.077283 |
| SYNCRIP | 78.27007 | 0.077283 |
| TMEM161A | 15.68976 | 0.077283 |
| ABT1 | 11.82661 | 0.084539 |
| NAA16 | 6.386986 | 0.085141 |
| TOP1MT | 29.52749 | 0.08967 |
| AGMAT | 19.78851 | 0.093821 |
| ATP5F1E | 294.1887 | 0.093821 |
| BRCA1 | 7.362393 | 0.093821 |
| CASP8AP2 | 3.736329 | 0.093821 |
| CCNE1 | 20.86825 | 0.093821 |
| CD81 | 64.67264 | 0.093821 |
| CDK5RAP1 | 7.306357 | 0.093821 |
| CGAS | 4.795412 | 0.093821 |
| CPNE1 | 59.11708 | 0.093821 |
| CXCL2 | 25.85707 | 0.093821 |
| DOHH | 20.91704 | 0.093821 |
| FAM217B | 35.17243 | 0.093821 |
| FKBP14 | 4.740999 | 0.093821 |
| GMEB2 | 6.160716 | 0.093821 |
| GNA12 | 15.42127 | 0.093821 |
| GPX7 | 7.038872 | 0.093821 |
| HBS1L | 9.920257 | 0.093821 |
| HPDL | 21.85322 | 0.093821 |
| ID1 | 5.745519 | 0.093821 |
| KIAA1143 | 8.996376 | 0.093821 |
| KIAA1586 | 3.300663 | 0.093821 |
| KIF2C | 27.44997 | 0.093821 |
| LIG3 | 6.361544 | 0.093821 |
| MCM4 | 22.08155 | 0.093821 |
| MRPL4 | 47.57655 | 0.093821 |
| NUP160 | 13.24277 | 0.093821 |
| PABPC4 | 18.86084 | 0.093821 |
| PLXNA1 | 6.835913 | 0.093821 |
| POMGNT2 | 6.69729 | 0.093821 |
| PTBP1 | 16.21029 | 0.093821 |
| RRM1 | 13.51825 | 0.093821 |
| SF3A3 | 13.66286 | 0.093821 |
| SNX10 | 3.228795 | 0.093821 |
| SRM | 60.46413 | 0.093821 |
| SSB | 54.1071 | 0.093821 |
| SSRP1 | 34.97398 | 0.093821 |
| UTP18 | 24.01524 | 0.093821 |
| WDR46 | 32.40529 | 0.093821 |
| XPO5 | 16.10685 | 0.093821 |
| ZHX3 | 4.409651 | 0.093821 |
| PTPN1 | 13.11177 | 0.09499 |
| TPX2 | 39.39598 | 0.09499 |
| ADNP | 15.42412 | 0.095754 |
| PA2G4 | 21.63976 | 0.096682 |
| MFSD12 | 18.93876 | 0.098061 |
| ORC6 | 2.490791 | 0.098061 |
| SNRPD2 | 316.4874 | 0.098061 |
| TP53RK | 22.63215 | 0.098061 |
| H4C3 | 194.0825 | 0.098338 |
| ANAPC7 | 10.05521 | 0.0996 |
| ATAD2 | 2.03133 | 0.0996 |
| GEMIN5 | 21.01518 | 0.0996 |
| NONO | 17.83236 | 0.0996 |
| SEC61G | 114.466 | 0.0996 |
| KIF11 | 14.80282 | 0.099708 |
| C19orf48 | 15.29424 | 0.099998 |
| DPP9 | 10.40221 | 0.099998 |
| SRSF6 | 14.33618 | 0.099998 |
| XRCC1 | 8.797703 | 0.099998 |

**Supplementary Table S2.** Result of over-representation analysis of the chemoradiosensitivity signature in the KEGG pathways.

| Term | *P*-value | Combined Score |
| --- | --- | --- |
| Base excision repair | 5.11E-04 | 163.742 |
| Glutathione metabolism | 1.56E-04 | 144.3636 |
| DNA replication | 6.62E-04 | 143.7541 |
| Cell cycle | 2.79E-05 | 118.5684 |
| RNA transport | 0.001943 | 37.79732 |
| Arginine and proline metabolism | 0.023592 | 33.33398 |
| IL-17 signaling pathway | 0.01019 | 32.56367 |
| Pyrimidine metabolism | 0.02912 | 27.95673 |
| TNF signaling pathway | 0.016301 | 24.37885 |
| p53 signaling pathway | 0.047195 | 18.34398 |
| Ubiquitin mediated proteolysis | 0.02913 | 16.63748 |

**Supplementary Table S3.** Result of over-representation analysis of the chemoradiosensitivity signature in the Gene ontology pathways.

| Term | P-value | Combined Score |
| --- | --- | --- |
| DNA replication | 8.23E-07 | 218.5275 |
| spliceosomal complex assembly | 6.69E-05 | 199.8218 |
| double-strand break repair via homologous recombination | 6.83E-06 | 174.589 |
| mRNA splicing, via spliceosome | 7.17E-07 | 123.8294 |
| DNA metabolic process | 7.92E-07 | 121.5621 |
| positive regulation of cAMP-mediated signaling | 0.032785 | 120.5864 |
| mRNA processing | 1.63E-06 | 106.0521 |
| cellular macromolecule biosynthetic process | 2.45E-06 | 97.99684 |
| base-excision repair | 0.001538 | 93.21549 |
| positive regulation of DNA repair | 0.001538 | 93.21549 |
| recombinational repair | 5.47E-04 | 87.30281 |
| double-strand break repair via nonhomologous end joining | 0.003206 | 62.99031 |
| double-strand break repair | 0.001116 | 46.90498 |
| nucleotide-excision repair | 0.013729 | 27.14845 |
| Notch signaling pathway | 0.033065 | 25.08867 |
