# Supplementary material for: Characterization of the gene signature correlated with favorable response to chemoradiotherapy in rectal cancer: A hypothesis‐generating study
Source: Cancer Med. 2023 Jan 9;12(7):8981–90. doi: 10.1002/cam4.5586 (PMC10134325; doi:10.1002/cam4.5586)
Supplement: Supplementary file 2 — Figure S1. Figure S2. [file CAM4-12-8981-s002.pdf]

Supplementary Figure S1

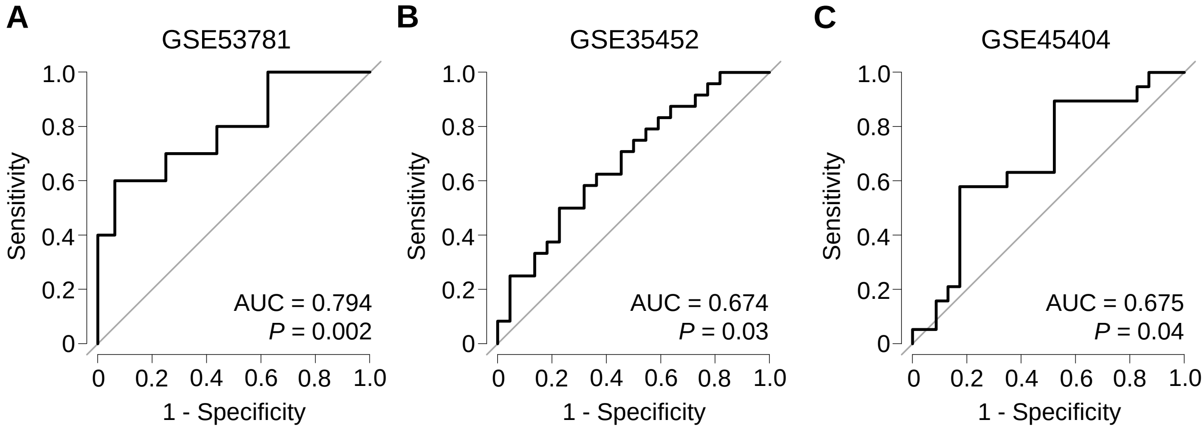

Supplementary Figure S1. Predictive performance of the chemoradiosensitivity (CRS) signature. (A-C) The ROC curves for the CRS signature in GSE53781 (A), GSE35452 (B), and GSE45404 datasets (C). *P*-values were calculated using Delong's test.

Supplementary Figure S2

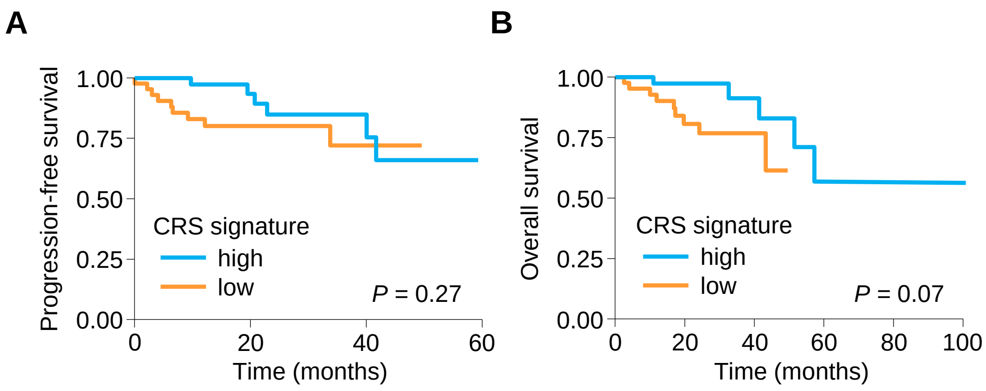

Supplementary Figure S2. Association between the chemoradiosensitivity (CRS) signature and patient outcomes. (A-B) Progression-free survival (A) and overall survival (B) according to CRS signature score in TCGA dataset. *P*-values were calculated using Cox-proportional hazard model.
